# Collaborative Prescribing Practice in Managing Patients Post-Bariatric Surgery in a Tertiary Centre in Singapore

**DOI:** 10.3390/pharmacy12010031

**Published:** 2024-02-08

**Authors:** Giat Yeng Khee, Paik Shia Lim, Yoke Ling Chan, Phong Ching Lee

**Affiliations:** 1 Department of Pharmacy, Singapore General Hospital, Singapore 169608, Singapore; lim.paik.shia@sgh.com.sg; 2 Department of Speciality Nursing, Singapore General Hospital, Singapore 169608, Singapore; chan.yoke.ling@sgh.com.sg; 3 Department of Endocrinology, Singapore General Hospital, Singapore 169608, Singapore; lee.phong.ching@singhealth.com.sg

**Keywords:** collaborative prescribing, bariatric surgery, pharmacist, nurse, endocrinologist, collaboration, obesity

## Abstract

**Background:** A collaborative prescribing (CP) practice model, established by the endocrinologists, pharmacists, and advanced practice nurses, aims to provide for the postoperative monitoring and medical and nutritional management of stable patients after bariatric surgery. **Method:** Under the CP agreement, endocrinologists refer patients who have undergone bariatric surgery with stable medical conditions to CP practitioners, comprising senior pharmacists and advanced practice nurses. CP practitioners review the patient’s weight loss progress, blood test results and vitals, the sufficiency of micronutrient repletion, adherence to supplements and medications, and chronic disease control. CP practitioners can prescribe and adjust the medications and supplements, in accordance with a clinical evaluation and standard guidance. Patients who require immediate attention due to complications or red flags are referred to the primary endocrinologist for further management. **Results:** From 5 May 2020 to 30 September 2023, CP practitioners provided 672 consultations. At least 68% and 80% of patients achieved appropriate weight loss post-surgery during the acute and maintenance phases, respectively. Less than 10% of the patients presented with anaemia and iron deficiency, and vitamin B12, folate and vitamin D deficiency. More than 80% of patients achieved a HbA1c of less than 7%. **Conclusions:** The CP practice framework provides a sustainable and viable model to facilitate optimal outcomes after bariatric surgery.

## 1. Introduction

Obesity is a chronic, progressive, and often relapsing [1,2,3] disease that is associated with complications such as diabetes, coronary heart disease, sleep apnoea and increased mortality [4]. The prevalence of obesity is increasing at an alarming rate in developing countries, including many parts of Asia [5]. Based on the 2019/2020 National Population Health Survey, the prevalence of obesity among adult Singaporeans aged 18 to 74 years was 10.5% [6]. The treatment of obesity includes lifestyle modifications, anti-obesity medications, and bariatric surgery. Evidence-based integrated approaches are warranted to help people with obesity lead healthier lifestyles, and achieve sustained weight loss, weight maintenance, and improved health. In Singapore, various strategies were adopted to combat obesity by promoting supportive environments, restricting energy-dense food to children, and allowing increased access to exercise and fitness facilities [7,8]. Bariatric surgery is recommended for patients with severe obesity, as it is the treatment option that provides the most significant and durable weight loss [9,10,11]. Sleeve gastrectomy (SG) and Roux-en-Y gastric bypass (RYGB) lead to an average weight loss of 25% and 30%, respectively [12]. Weight loss is also sustained after bariatric surgery, with an average weight regain of 5% to 10%, as documented from the nadir weight after 10 years [13,14]. In addition, weight loss following bariatric surgery results in the significant improvement and resolution of associated medical conditions such as type 2 diabetes mellitus, hypertension, and obstructive sleep apnoea [13,15,16,17]. The American Diabetes Association 2023 Guideline recommends that bariatric surgery should be considered to treat type 2 diabetes in adults with a body mass index (BMI) ≥ 40 kg/m^2^ (37.5 kg/m^2^ in Asian individuals) or in adults with a BMI of 35.0–39.9 kg/m^2^ (32.5–37.4 kg/m^2^ in Asian individuals) who do not achieve durable weight loss and improvements in co-morbidities with nonsurgical methods [18]. The number of bariatric surgeries performed has grown significantly over the years, in Singapore and worldwide. Our obesity centre operates the largest bariatric surgery programme in Singapore, with at least 150 bariatric surgeries performed annually and 1500 patients who have had bariatric surgeries at our centre. The two most performed bariatric surgeries in the obesity centre are the SG and RYGB.

Despite its clinical benefits, bariatric surgery is associated with long-term risks including nutritional deficiencies and weight regain [19,20,21]. There have been case reports of nutritional deficiencies leading to cardiomyopathy, night blindness, and neuropathy, including permanent disability or death in some cases [22,23,24,25,26]. Nutritional deficiencies can occur due to modifications to gastrointestinal anatomy and physiology, which could affect macro- and micro-nutrient absorption [27,28,29]. The commonly cited contributing factor was inadequate follow-up or adherence to supplements. Weight regain was observed to typically begin in the second postoperative year [30]. Cohort studies and systematic reviews have demonstrated that poor follow-up care and adherence to supplements have negative impacts on outcomes [31,32,33]. With the high prevalence of nutrient deficiencies and potential weight regain, clinical guidelines recommend long-term regular lifestyle support, medical monitoring, and management for people undergoing bariatric surgery for positive postoperative outcomes and improved quality of life [10,34,35,36,37]. The National Institute for Health and Care Excellence and the European Association for the Study of Obesity suggest that long-term care can be delivered in primary care, under a shared care model, in conjunction with a bariatric specialist [10,37]. In Singapore, long-term post-bariatric follow-up care is provided by the obesity specialist in tertiary hospitals. To ensure adequate follow-up and positive outcomes with bariatric surgery for this growing population, we adopted the strategy of integrating collaborative prescribing (CP) practitioners in the shared care model.

In Singapore, CP is a new professional practice area for both advanced practice nurses and senior pharmacists, in which they are upskilled to legally prescribe medicines, without the need to obtain a doctor’s countersignature and order laboratory investigation tests in a collaborative framework overseen by doctors [38,39,40]. CP is part of Singapore Ministry of Health’s care transformation effort in the community and hospitals, providing holistic and better continuity service through team-based care [40]. Under the national CP programme, CP practitioners are trained to possess competencies and skills in history taking, data interpretation, diagnostic formulation, physical examination, clinical decision making, applied therapeutics, psychosocial aspects of prescribing, collaboration with multidisciplinary teams, effective communication, and documentation [38,39,40].

The CP model aims to increase patients’ accessibility to healthcare while empowering advanced practice nurses and senior pharmacists to perform advanced roles and increase workforce productivity [40]. Such a model also helps to alleviate doctors’ time constraints while facilitating patients’ adherence to the ongoing support provided by CP practitioners between doctor consultation visits. This could potentially reduce or even prevent delays in the treatment and management of disease conditions, and ultimately improve patient outcomes. The CP clinic Obesity Management Clinic (OMC) was established in 2020. The purpose of this paper is to describe the CP care model in managing a post-bariatric surgery population in an obesity centre. 

## 2. Materials and Methods

### 2.1. Development of Collaborative Prescribing Agreement (CPA)

Prior to the establishment of the CP practice in the obesity centre, the advanced practice nurse and senior pharmacists were working in team-based practice with the Department of Endocrinology. Team-based practice includes endocrine ward rounds; an inpatient glucose management service; and a young adults with diabetes clinic. Obesity management is a new practice area for both the advanced practice nurse and senior pharmacists. Shadowing the multidisciplinary team in the obesity centre enabled transdisciplinary learning for the advanced practice nurse and senior pharmacists, and the co-development of the CPA with the endocrinologist.

A CPA is a formal agreement between CP practitioners and their collaborating doctor, endorsed by clinical and professional heads of department, and approved by a healthcare institution’s credentialing committee [39]. This agreement specifies the CP practitioner’s scope of practice, which includes the following: medical conditions and/or defined patient groups, drug formulary, clinical decisions, tests and investigations, escalation criteria, and patient exclusion criteria [39]. The clinical governance framework was established in accordance with the Guidelines for The Implementation of CP Services, by the Ministry of Health, Singapore [39]. Standard guidance for postoperative clinical and biochemical monitoring and micronutrient supplementations were included in the CPA, with reference to the Bariatric Surgery Guidelines [34].

The CPA was subsequently presented, discussed, and refined during meetings with the obesity multidisciplinary team, the department of pharmacy, and the nursing division. The CPA was reviewed and approved by the hospital CP Credentialing Committee in 2019.

### 2.2. Scopes of Practice of OMC CP Practitioners

**Figure 1:**
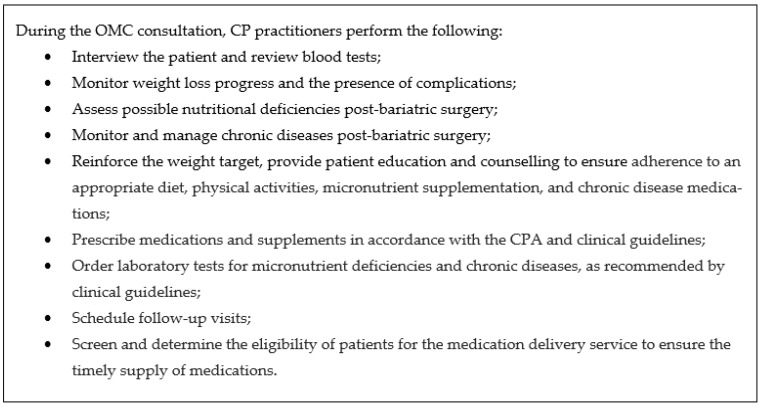
Scopes of practice of OMC CP practitioner.

The objectives of the CP practice in OMC are to provide for the long-term postoperative monitoring and medical and nutritional management of patients after bariatric surgery. The endocrinologists in the obesity centre refer patients who underwent RYGB and SG with stable chronic medical conditions, to the OMC. CP practitioners practice under the supervision of the attending endocrinologist, who would be available for discussion, if required. The scopes of practice of the OMC CP practitioner are illustrated in Figure 1.

### 2.3. Post-Bariatric Surgery Patient’s Journey in a CP Model

**Figure 2:**
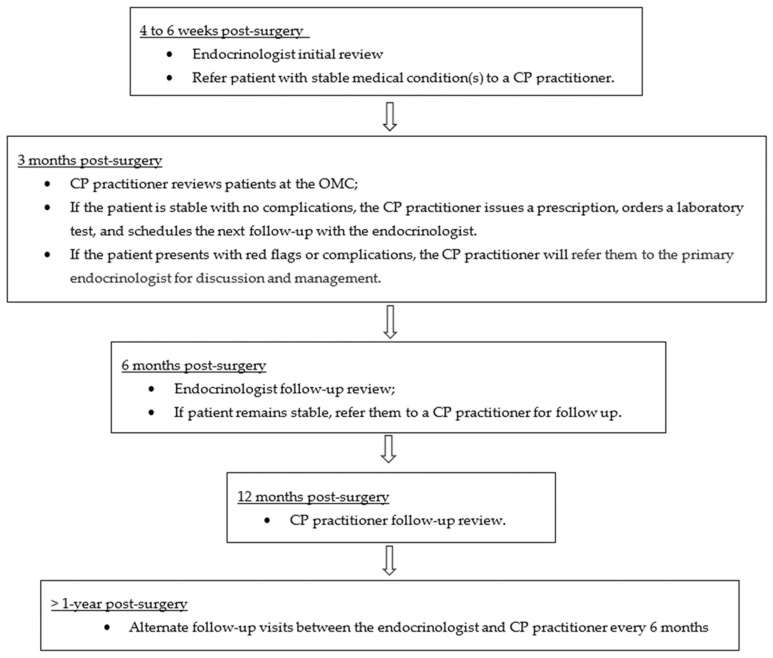
OMC visit algorithm.

**Figure 3:**
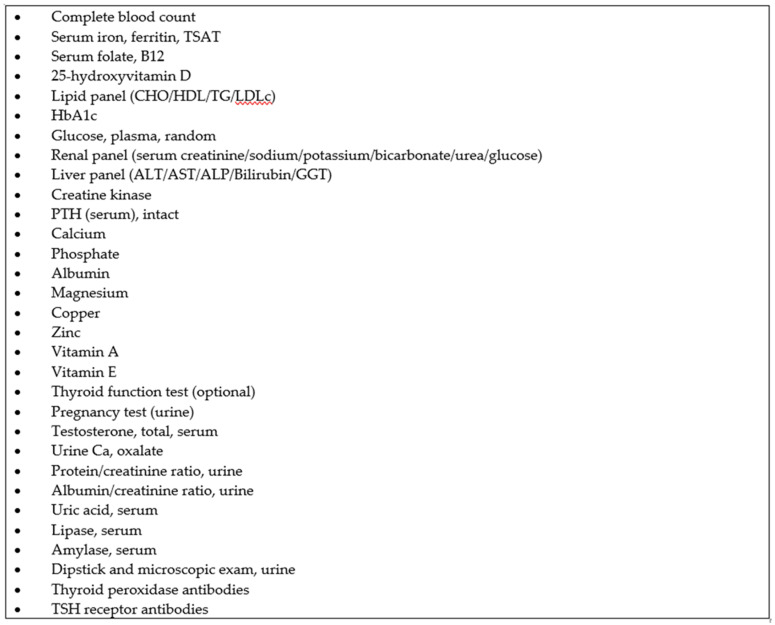
Laboratory investigations listed in the CPA.

**Figure 4:**
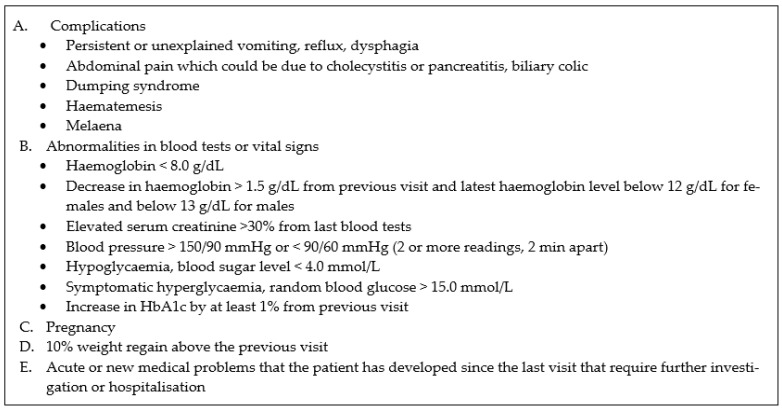
Escalation criteria for referral to an endocrinologist.

Referred patients are followed up in accordance with the OMC visit algorithm in Figure 2. At 4 to 6 weeks after surgery, patients are reviewed by an endocrinologist. If a patient has a stable medical condition, the endocrinologist can refer the patient to the OMC for follow-up 3 months after surgery. During the OMC appointment, the CP practitioner will review the patient’s weight, most recent blood test results, and vitals to assess the sufficiency of micronutrient repletion and chronic disease control, and enquire about adherence to the micronutrients and chronic disease medications given. Standard measurement such as blood pressure and heart rate are performed routinely. The CP practitioner can prescribe and adjust the medications and supplements, based on the clinical evaluation and standard guidance. Laboratory tests for micronutrient deficiencies and chronic diseases can be ordered in accordance with the CPA (Figure 3). Patients who require immediate attention due to complications or red flags, which are listed under escalation criteria in the CPA, are referred to the primary endocrinologist immediately for discussion and management (Figure 4). Patients are scheduled to be reviewed by the primary endocrinologist after 3 months. Patients have alternate visits between the endocrinologist and CP practitioner every 3 months during the first year after bariatric surgery. Subsequently, the return visit interval is extended to every 6 months, and patients have alternate visits between the endocrinologist and CP practitioner.

### 2.4. Quality and Safety Monitoring

To ensure safe and effective CP practice, the Singapore Ministry of Health establishes two levels of safeguards [40]. Firstly, only advanced practice nurses and senior pharmacists who are trained under the 13-week National Collaborating Prescribing Programme provided by the National University of Singapore can be qualified to prescribe [40]. A doctor, who takes on the role of clinical supervisor, will provide supervision, support, and opportunities to develop one’s competency in prescribing practice [40]. Secondly, healthcare institutions offering CP services are required to set up a credentialing committee that will verify and approve the participating nurses, pharmacists and their collaborative services, and a Service Review Committee that will monitor, audit, and review these prescribing services to ensure effectiveness and safety [39].

Clinic indicators for the OMC of safety, process, outcome, and quality are monitored, audited, and reviewed on a yearly basis. The categories and definitions of the indicators and targets are illustrated in Table 1. The CP practitioners and collaborating endocrinologist should discuss and formulate improvement plans based on a review of the service performance indicators.

### 2.5. Competency Maintenance for CP Practitioner

The competency maintenance requirements that need to be fulfilled by the CP practitioner include a specific number of OMC sessions, the attendance of obesity-related continuing education, and the completion of prescribing logs. The CP practitioner attends obesity team meetings and conferences fortnightly for regular updates and to learn of the latest developments and best practices in the field of obesity. In addition, regular meetings are organised between the collaborating endocrinologist and CP practitioners to discuss cases and challenges encountered while providing the CP service.

## 3. Results

**Figure 5:**
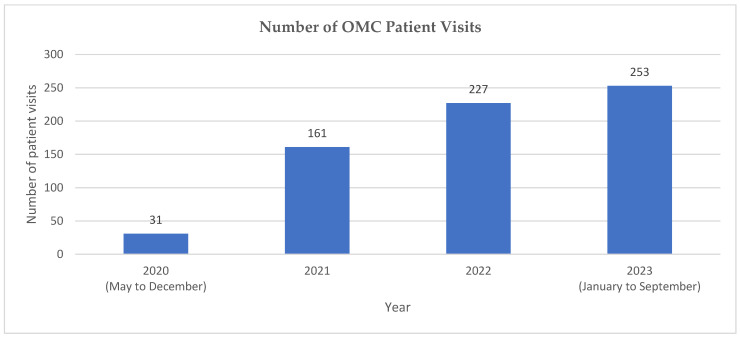
Number of patient visits at the OMC.

From 5 May 2020 to 30 September 2023, the OMC’s CP practitioners provided 672 consultations, in which 633 and 39 were face-to-face and virtual consultations, respectively. The number of referrals made by the endocrinologists to the OMC has increased steadily from 2020 to 2023, from an average of 3 to 4 patients a month to 28 patients a month (Figure 5). All of the OMC’s CP practitioners met the process indicator by providing at least 30 consultations a year. No errors or near-misses in prescribing, laboratory test ordering, or CP agreement deviations were reported. During the acute phase of post-bariatric surgery, which is defined as being after one-year post-surgery, 68% of patients show weight loss of at least 20% for SG and 25% for RYGB. During the chronic phase of post-bariatric surgery, which is defined as being after one-year post-surgery, 80% of patients show a weight regain of less than 10%, in relation to the nadir weight post-surgery. The proportion of patients with anaemia and ferritin deficiency, vitamin B12 deficiency, folate deficiency, and vitamin D deficiency was 6.3%, 4.9%, 6.3%, and 1.3%, respectively. At least 88% of patients achieved a HbA1c of less than 7%. The OMC has achieved the targets for all service performance indicators, as shown in Table 1.

There were 123 CP consultations that fulfilled the criteria stated in Figure 4 and that required escalation to the referring endocrinologist for a discussion on the management plan. The most common reasons for escalation include abnormalities in blood tests or vital signs, followed by acute or new medical problems, complications, pregnancy, and 10% weight regain above the previous visit (Table 2). The types of therapy adjustment made in the OMC include the initiation or adjustment of micronutrient supplements, chronic disease medications, weight loss medications, and moving appointments forward for early review and referral to specialists of other disciplines (Table 3).

## 4. Discussion

The CP practice model, established in a tertiary centre in Singapore, provides access to regular follow-up for the growing population of stable patients after bariatric surgery. The weight loss progress, surgical complications, and adherence to supplements and medications, chronic disease therapy can be monitored and managed in a timely manner. While CP practitioners manage postoperative patients with stable medical conditions, endocrinologists are able to focus on those with more complex medical conditions (not medication conditions). The number of referrals to the OMC has increased steadily since 2020, and the OMC has achieved the target for process, safety, outcome, and quality service performance indicators. Such a practice model facilitates the adequate follow-up and monitoring of patients post-bariatric surgery to minimise the risk of weight regain and micronutrient deficiency.

To our best knowledge, we are among the first hospitals in the world to include a pharmacist, advanced practice nurse, and endocrinologist in CP practice in an obesity centre. The available published literature on pharmacist- or nurse-led services in post-bariatric surgery care has mainly focused on medication reconciliation and reviews to reduce adverse events, weight loss progress, and blood pressure and capillary blood sugar monitoring [41,42,43,44]. This paper describes the development and implementation of the CP model in a well-defined population with clear pre-defined protocols in an obesity centre, to increase patients’ access to adequate follow-up on weight-loss progress, nutritional status, and co-morbidity management.

The driving forces behind the global development of prescribing roles for nurses and pharmacists are reported as increased access to healthcare services, heavy doctor workloads, better perceived patient outcomes, and the better use of pharmacists’ and nurses’ skills and knowledge for greater job satisfaction [45,46,47]. The CP model allowed for the increased accommodation of the needs of the expanding pool of post-bariatric surgery patients to receive timely and regular follow-up on weight loss progress, nutritional, and metabolic status. By leveraging the better use of skills within the multidisciplinary team, the CP model provides opportunities to further expand obesity management services in the obesity centre.

Patients with obesity often present with other metabolic complications and take multiple chronic medications such as anti-hypertensives, glucose, or lipid-lowering agents. Bariatric surgery can result in significant metabolic improvements such as reductions in blood pressure and HbA1c [48]. Data from our bariatric cohort showed that in patients with type 2 diabetes and severe obesity, 81% had a reduction in the number of diabetes medications one year after surgery [49]. Similarly, the use of blood-pressure-lowering medications declined from 84% at baseline to 40% at one year, and lipid-lowering medication usage declined from 78% at baseline to 37% at one year [49]. Hence, CP practitioners play a pivotal role in the close monitoring of blood pressure, and blood glucose and metabolic parameters after surgery, and make necessary adjustments to patients’ chronic medications to avoid complications such as hypotension and hypoglycaemia.

CP practitioners are also equipped with knowledge and skill sets that are in synergy with the skill sets required in diabetes management, which is a common complication of obesity. These competencies are acquired while they provide existing endocrinology services such as in the inpatient glucose management service and the young adults with diabetes clinic. Given their strong foundation in pharmacology, senior pharmacists are well placed to look out for any medication-related issues and potential drug–drug or drug–supplementation interactions during patient encounters. In the area of diabetes management, advanced practice nurses are well-trained in providing holistic care that includes regular glucose monitoring, medication titration, and foot and eye screening.

Micronutrient deficiencies are also highly prevalent in patients undergoing bariatric surgery. We previously reported on the prevalence of nutritional deficiencies in our bariatric cohort, with vitamin D, folate, iron, and vitamin B12 deficiencies being the most common deficiencies prior to surgery [27]. Anaemia is the most common nutritional complication after bariatric surgery, occurring in up to two-thirds of patients [50,51]. Data from our centre with a follow-up of up to 5 years showed that haemoglobin levels were significantly attenuated after bariatric surgery and never recovered to pre-bariatric procedure levels [29]. Our clinical findings above have led to the monitoring of vitamin D, folate, iron, and B12 deficiencies as key indicators of the OMC service. CP practitioners are well trained to review nutritional labs, and have made frequent adjustments to nutritional supplements, including the initiation of intravenous iron infusions for patients with iron deficiency anaemia refractory to oral therapy.

Although bariatric surgery is effective in producing long-term weight loss, there is wide variability in weight loss among individual patients. In our centre, centile charts are used for the monitoring of weight trajectories postoperatively and to facilitate realistic and personalised goal setting [52]. CP practitioners were trained to ensure that weight loss progress was appropriate. They play an important role in the early identification of “poor responders”, explore reasons for inadequate weight loss, and implement appropriate interventions. Endocrinologists, who were the clinical supervisors for CP practitioners during the National CP Programme, continue to mentor and supervise CP practitioners after the completion of the programme. Addressing all needs during a OMC consultation provides fresh challenges for CP practitioners within a safe learning environment to gain experience and build trust. The extension of prescribing rights to the CP practitioners not only recognises the capabilities of CP practitioners, but also provides them the autonomy to manage patients, which is an important milestone in materialising true inter-disciplinary patient care [39].

With the successful pilot implementation of the CP model in the obesity centre, the team plans to increase the number of clinic sessions and expand the patient population to include non-surgical patients with obesity who take anti-obesity medications, as well as managing pre-bariatric surgery population. The OMC’s CPA will be regularly reviewed and revised to accommodate the growing needs of the patient population, laboratory ordering, and drug formulary. A substantial body of evidence has suggested that CP practice is safe and acceptable [46,47]. Future plans for the OMC include evaluating the effectiveness and safety of the CP model with an expansion of its scope and the identification of opportunities for care model improvement.

## 5. Conclusions

This paper illustrates the development of a CP practice model by an advanced practice nurse, pharmacist, and endocrinologist, in the management of a post-bariatric surgery population in an obesity centre. The establishment of an OMC allowed for the increased accommodation of patients’ needs in postoperative care, with continued lifestyle counselling, weight loss progress and nutrition monitoring, and adjustments to therapy. This novel service integrates well into our multidisciplinary team’s approach toward optimal outcomes after bariatric surgery.

## Figures and Tables

**Table 1:** Clinic indicators for the OMC.

Category	Indicators	Target
**Process**	Number of patient visits per year	≥30
**Safety**	Number of errors or near-misses in prescribing or laboratory test ordering Number of CP agreement deviations	No event No event
**Outcome**	Appropriate weight loss post-surgery Acute phase: at one-year post-surgery, proportion of patients with weight loss at least 20% for SG and 25% for RYGB Maintenance phase: after one-year post-surgery, proportion of patients with weight regain of less than 10%, in relation to the nadir weight after surgery	≥50% ≥30%
**Quality**	Proportion of patients with nutritional deficiency and disease control: Anaemia (haemoglobin level < 12 g/dL in women and <13 g/dL in men) and ferritin <50 μg/L Vitamin B12 deficiency (vitamin B12 < 145 pmol/L) Folate deficiency (folate < 13.4 nmol/L) Vitamin D deficiency (vitamin D level < 20 ng/mL) Well-controlled diabetes (HbA1c < 7%)	<10% <10% <10% <10% >80%

**Table 2:** CP consultation requiring escalation to referring endocrinologist.

Criteria for Escalation to Referring Endocrinologist	Number of Consultation
Abnormalities in blood tests or vital signs	66
Acute or new medical problems since the last visit that require further investigation or hospitalisation	35
Complications	12
Pregnancy	6
10% weight regain above the previous visit	4
**Total**	123

**Table 3:** Types of therapy adjustment made in the OMC.

Types of Therapy Adjustment	Number
Micronutrient supplement initiation/adjustment	195
Others (starting or stopping medications or meal replacement, ordering additional investigations for acute medical conditions, etc.)	73
Chronic disease medication initiation/adjustment	51
Weight loss medication initiation/adjustment	23
Intravenous iron infusion initiation	21
Bringing forward appointments for early review	19
Referral to specialists of other disciplines or the emergency department	11
**Total**	393

## Data Availability

The datasets generated or analysed as part of this study are included in this published paper.
